# Percutaneous Treatment of Traumatic A3 Burst Fractures of the Thoracolumbar Junction Without Neurological Impairment: The Role of Timing and Characteristics of Fragment Blocks on Ligamentotaxis Efficiency

**DOI:** 10.3390/jcm14082772

**Published:** 2025-04-17

**Authors:** Mario De Robertis, Leonardo Anselmi, Ali Baram, Maria Pia Tropeano, Emanuela Morenghi, Daniele Ajello, Giorgio Cracchiolo, Gabriele Capo, Massimo Tomei, Alessandro Ortolina, Maurizio Fornari, Carlo Brembilla

**Affiliations:** 1 Department of Biomedical Sciences, Humanitas University, Via Rita Levi Montalcini 4, 20072 Pieve Emanuele, Italy; mario.derobertis@humanitas.it; 2 Department of Neurosurgery, IRCCS Humanitas Research Hospital, Via Manzoni 56, 20089 Rozzano, Italy; ali.baram@humanitas.it (A.B.); maria.tropeano@humanitas.it (M.P.T.); gabriele.capo@humanitas.it (G.C.); massimo.tomei@humanitas.it (M.T.); alessandro.ortolina@humanitas.it (A.O.); maurizio.fornari@humanitas.it (M.F.); carlo.brembilla@humanitas.it (C.B.); 3 Biostatistics Unit, IRCCS Humanitas Research Hospital, Via Manzoni 56, 20089 Rozzano, Italy; emanuela.morenghi@humanitas.it; 4 Neuroradiology Department, IRCCS Humanitas Research Hospital, Via Manzoni 56, 20089 Rozzano, Italy; daniele.ajello@humanitas.it; 5 School of Medicine and Surgery, Pope John XXIII Hospital, University of Milano-Bicocca, 24127 Bergamo, Italy; g.cracchiolo@campus.unimib.it

**Keywords:** fracture, thoracolumbar, ligamentotaxis, percutaneous, timing

## Abstract

**Background:** This study aims to evaluate how surgical timing and the radiological characteristics of fragment blocks can affect the effectiveness of ligamentotaxis, in restoring the spinal canal area, and local kyphosis in adults with traumatic thoracolumbar A3 burst fractures without neurological impairment treated with percutaneous short-segment fixation. **Methods:** A retrospective observational study was conducted between January 2016 and December 2022 on neurologically intact adult patients with a single A3 thoracolumbar fracture. Data collected included demographics, injury mechanism, fracture level, and clinical and surgical details. Radiological assessments included spinal canal area, local kyphotic angle, anterior and posterior vertebral heights, and fragment block measurements. **Results:** Out of 101 treated patients, 9 met the criteria with a mean age of 52.22 years. Most fractures were at L1 (88.89%). All patients had moderate-to-severe pain (NRS 6.22 ± 1.09) at baseline. Five patients (55.55%) underwent surgery within 72 h, with a mean surgical time of 109.22 min. SCA and LKA values improved significantly in all patients post-surgery. Early surgical intervention (<72 h) correlated with greater improvements in spinal canal area (*p* = 0.016) and local kyphotic angle (*p* = 0.004). A significant association was found between spinal canal area improvement and the percentage ratio of fragment height to “normal” vertebral height (rho = 0.682; *p* = 0.043). **Conclusions:** Early (<72 h) short-segment percutaneous fixation is recommended for adults with high functional demands and moderate-to-severe axial pain due to single traumatic A3N0M0 thoracolumbar fracture. This “upfront” approach is associated with enhanced indirect decompression and better local kyphotic angle restoration. Considering the fragment morphology could also be important in surgical planning.

## 1. Introduction

The thoracolumbar (TL) junction (T10-L2) is particularly susceptible to high-energy forces due to its unique transitional anatomy, shifting from a stiff kyphotic region to a more lordotic and mobile one.

The most common causes of thoracolumbar fractures are falls and traffic accidents, with an annual incidence of approximately 30 per 100,000 inhabitants, including osteoporotic fractures. However, the true incidence and epidemiology in developing countries remain poorly understood [1].

Burst fractures are particularly common at the T12-L1 level, accounting for nearly 20% of thoracolumbar trauma cases [2]. These fractures result from the compressive failure of anterior and middle Denis columns. The extent of spinal canal stenosis due to the retropulsion of the posterior wall fragment varies, potentially leading to neurologic impairment.

The optimal surgical management of thoracolumbar burst fractures requires a comprehensive evaluation of the patients’ neurological status, pain control, the degree of kyphotic deformity, the extent of spinal canal stenosis and/or instability on imaging, and overall injury burden in polytrauma cases [3].

Traumatic A3 fractures (according to the AO Spine Classification) [4], especially in the absence of neurological deficits, represent a grey zone, with multiple treatment options ranging from conservative management to surgical fixation, depending on the surgeon’s preference and the patient’s clinical setting [3,5,6,7].

For burst fractures with retropulsed fragments (with no neurological deficits), a minimally invasive posterior approach can achieve effective fracture reduction through ligamentotaxis, thereby restoring spinal alignment and stability, reducing pain, and facilitating an early return to daily activities [3,6]. Although early surgery (within 24 h) is generally recommended for patients with neurological impairment, the precise timing cutoff lacks uniform consensus [8]. For neurologically intact patients, there are no data in the literature regarding the optimal timing of surgical intervention, particularly regarding the effectiveness of vertebral restoration.

The aim of this study is to assess the impact of surgical timing and the radiological characteristics of fragment blocks on the efficiency of ligamentotaxis and the improvement of local kyphosis in a highly selected cohort of adult patients with traumatic thoracolumbar A3 (AO Spine Classification) burst fractures without neurological impairment, treated with percutaneous short-segment fixation.

## 2. Materials and Methods

### 2.1. Study Design

This is a monocentric, retrospective, observational study of adult patients with traumatic burst fractures of the thoracolumbar junction, surgically treated between January 2016 and December 2022. Informed consent was obtained from all patients prior to the surgical procedures. The study was approved by our institutional ethics committee (N° 10/24). Inclusion criteria were as follows: patients aged 18 to 65 years, presenting with a single A3 type fracture (according to the AO Spine Classification) [4] at the thoracolumbar junction (T10 to L2), resulting from high-energy trauma, with an absence of neurologic deficits (classified as grade E according to the ASIA classification), and treated with short-segment percutaneous posterior fixation.

Exclusion criteria included osteoporotic fractures (patients with a clinical history of osteoporosis/osteopenia AND a positive DEXA), pathological fractures, fractures outside the thoracolumbar junction, and a lack of adequate clinical and radiological follow-up at 12 months after surgery.

### 2.2. Patient Sample

General patients’ data, including age, gender, and BMI, were recorded. Clinical data such as neurological status based on the ASIA scale and NRS back pain scores at admission and discharge were collected and compared before and after surgery. Additional information gathered included the mechanism of injury, fracture level, the presence of other bone and/or visceral injuries, and intra- and post-operative adverse events which were graded according to the Spinal Adverse Events Severity System, version 2 (SAVES v-2) [9]. An arbitrary cutoff of 72 h was used to categorize the population in early (<72 h) versus late (>72 h) surgery groups. Surgical data, including the duration of surgery and the number of levels fixed (considering the presence or absence of pedicle screws at the fractured site), were collected from operative reports. All patients were graded according to Thoracolumbar Injury Classification System (TLICS) [10] and Load-Sharing Classification (LSC) [11] scores.

### 2.3. Radiological Data

In all patients included in the study, a pre-operative thoracolumbar spine CT scan was performed regardless of the timing of the traumatic event. Additionally, a thoracolumbar spine MRI was performed to complete the pre-operative assessment. The aim was to evaluate the integrity of the posterior ligamentous complex (PLC), particularly the posterior longitudinal ligament (PLL) (according to Grenier et al.) [12], and to identify other potential injuries not detectable by CT. In our practice, follow-up assessments included a post-operative thoracolumbar spine CT scan performed at 12 months after surgery to verify vertebral bone healing, associated with an outpatient clinical evaluation. While the lack of upright imaging presents a limitation in assessing the impact on kyphosis restoration, the choice of CT scan analysis lies in the necessity of methodological homogeneity in parameter comparison and in the possibility of a higher-quality detail imaging evaluation, especially on the axial plane. Radiological data were collected and analyzed by a single radiologist of our center, comparing the pre-operative and follow-up CT scan in the 3 planes (software IntelliSpace Portal v. 10—Philips).

**Figure 1:**
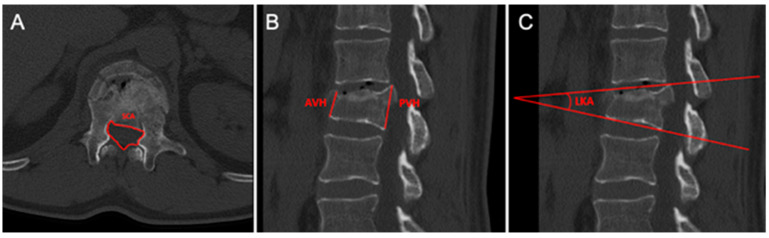
CT general radiological parameters. (**A**) SCA (spinal canal area). (**B**) AVH (anterior vertebral height) and PVH (posterior vertebral height). (**C**) LKA (local kyphotic angle).

General radiological parameters (Figure 1):

- Spinal canal area (SCA): the area of the spinal canal at the level of maximum bone fragment retropulsion.
- Anterior vertebral height (AVH) (mm): the distance between the antero-superior and antero-inferior edge of the fractured vertebra, measured at the level of the anterior vertebral body wall.
- Posterior vertebral height (PVH) (mm): the distance between the postero-superior and postero-inferior edge of the fractured vertebra, measured at the level of the posterior vertebral body wall.
- Local kyphotic angle (LKA): the angle formed by a line drawn along the superior end plate, considering the apex of the protruded fragment, and the inferior end plate of the fractured vertebra.

The difference between post-operative and pre-operative CT parameters (delta values) was considered for the statistical analysis.

**Figure 2:**
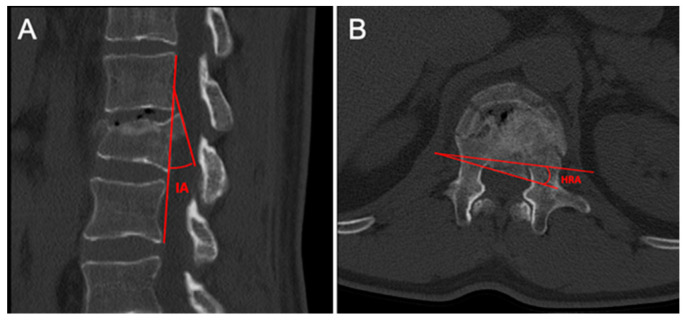
CT bone fragment parameters. (**A**) IA (inversion angle). (**B**) HRA (horizontal rotation angle).

Bone fragment measurement (Figure 2).

The parameters related to the retropulsed bone fragment of the fractured vertebra’s posterior wall were as follows:

- Position of the fragment block: evaluated on the axial plane at CT scan; the posterior wall of the fractured vertebra at the level of the spinal canal was divided into three equal sectors, defining the fragment as lateral (left or right), paramedian (middle-left/-right), or median.
- Inversion angle (IA): measured on the sagittal plane at the point of maximum protrusion of the fragment, it is defined as the intersection angle between the tangent line to the “ideal” posterior vertebral wall and the line to the posterior margin of the bone fragment.
- Horizontal rotation angle (HRA): measured on the axial plane at the point of maximum stenosis, it is defined as the intersection angle between the line tangent to the posterior “ideal” vertebral wall and the line to the posterior margin of the intracanalar fragment.
- % ratio of fragment/vertebra height: the ratio between the height of the bone fragment, measured at its maximum diameter on the sagittal plane, and the assumed “normal” height of the injured vertebra for the individual patient, calculated as the average heights of the vertebrae above and below the site of injury.
- % ratio of fragment/vertebra width: the ratio between the width of the bone fragment, measured at its maximum diameter on the axial plane, and the assumed “normal” transverse diameter of the vertebral canal of the injured vertebra for the individual patient, calculated as the average transverse diameters of the vertebrae above and below the site of injury.

### 2.4. Surgical Technique

All patients underwent short-segment fixation, involving one level above and one level below the fracture site, using a percutaneous posterior approach. In all patients, polyaxial screws were adopted. The procedure was performed with the use of an intraoperative 3D-CT scan interfaced with a Neuronavigation System (O-ARM Treon Medtronic). Imaging was acquired both before and after the placement of pedicle screws to facilitate navigation and to verify the correct placement, respectively. The same percutaneous fixation hardware was used for all patients, and surgery was performed by the same surgical team. If the pedicles of the fractured vertebrae were intact, screw insertion was carried out at this level (in one of the two pedicles), to enhance the construct strength. After contouring the rods, reduction maneuvers were performed. Specifically, if fixation involved only the upper and lower vertebra, the two levels were distracted “monoaxially”. When screws were also positioned in the fractured level, the “monoaxial” distraction involved the fractured vertebra and the one above it. Finally, polyaxial compression was carried out between the upper and lower vertebra, to partially restore the AVH and LKA of the injured site. No fusion was performed on any of the patients.

### 2.5. Statistical Analysis

Data are described as number and percentage, if categorical, or mean and standard deviation, if continuous. Only the duration of recovery is described as median and range, to underline minimum and maximum value of this variable. Variation in NRS is described as mean, standard deviation, and 95% confidence interval. Associations between continuous variables were explored with Pearson coefficient rho, and a value over 0.6 was considered clinically interesting. Differences between pre- and post-surgery parameters were explored with the Wilcoxon test. All analyses were performed with Stata version 18.

## 3. Results

Between January 2016 and December 2022, 101 patients with traumatic vertebral fracture were treated at the Department of Neurosurgery of Humanitas Research Hospital, after admission from the Emergency Department (ED). Of these, 52 patients were excluded because they were treated with an open posterior approach, leaving 49 patients treated with a percutaneous approach. From this group, 40 patients were further excluded: 33 due to lack of radiological follow-up available, 1 due to age > 65 years, 2 with multiple vertebral fractures, 2 with fractures in T7 and L3, and 2 with different fracture types (B2 and A2).

The final study population included nine patients (five M and four F), with a mean age of 52.22 (±13.73) and a mean BMI of 27.66 (±3.96) (Table 1).

None of patients had major comorbidities affecting the decision-making process.

All patients had traumatic incomplete burst fractures of the thoracolumbar junction (A3, according to AOSpine Classification), with eight fractures at L1 (88.89%) and one at L2 (11.11%). Seven patients experienced falls from a height > 3 m (77.78%) and two were involved in high-energy road traffic accidents (22.22%). Two patients had additional fractures in other bone sites, which were treated subsequently, and none had associated visceral lesions.

All patients had a TLICS score of 2. Regarding the MacCormack score (Load-Sharing Classification), two patients (22.22%) had a score of 8, two (22.22%) had a score of 7, three (33.33%) had a score of 6, and two (22.22%) had a score of 4, with a median LSC score of 6 (range 4–8). All patients had moderate-to-severe back pain at presentation. Five patients (55.55%) were treated within 72 h of trauma. In the other four (44.44%), patients an initial attempt of pain medication strategy failed and required ED access.

**Figure 3:**
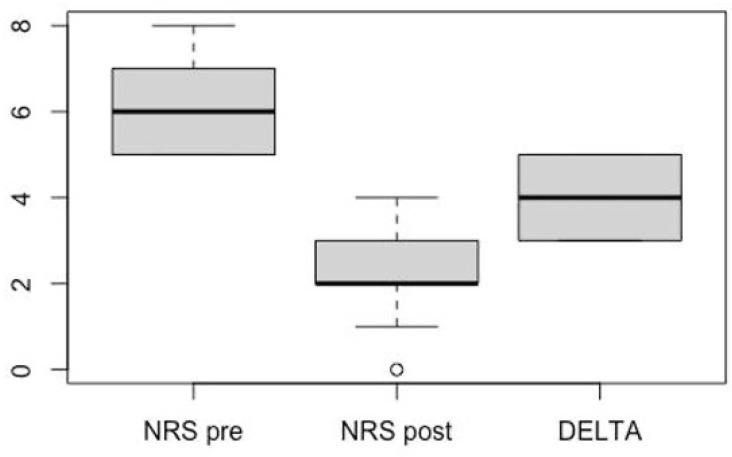
Boxplot of pre- and post-operative NRS score variation.

The NRS back pain score improved from a mean of 6.22 (±1.09) at admission to a mean of 2.22 (±1.20) at discharge (*p* = 7.115 × 10^−7^), with patients rapidly returning to normal daily activities without the need for a brace (Table 1 and Figure 3). The neurological status, reported as ASIA E at baseline, remained unchanged after surgery.

In addition, in the early surgery group, four out of five patients resumed work within 2 weeks, and one patient resumed work after 3 weeks post-operatively. In contrast, in the late surgery group, three out of four patients returned to work after 4 weeks, and one patient returned to work after 6 weeks post-operatively.

All patients were treated using a percutaneous posterior approach. In four cases, no screws were placed at the fractured level (44.44%), while in three cases, the fractured vertebra was fixed bilaterally (33.33%), and in one case (11.11%), it was fixed unilaterally within the intact pedicle. The mean surgical time was 109.22 (±16.69) minutes (Table 1). No patients required blood transfusions. The median length of stay was 5 days (range 2–17). Most patients had a regular post-operative course without adverse events. One patient developed a post-operative UTI (urinary tract infection, grade 2 according to SAVES v-2), resulting in a length of stay (LOS) increase of 4 days; another patient developed UTI and SIADH (syndrome of inappropriate antidiuretic hormone ADH release, grade 2 SAVES v-2), resulting in an LOS increase of 10 days.

We also considered the radiological parameters of the fragment block (Table 1).

**Figure 4:**
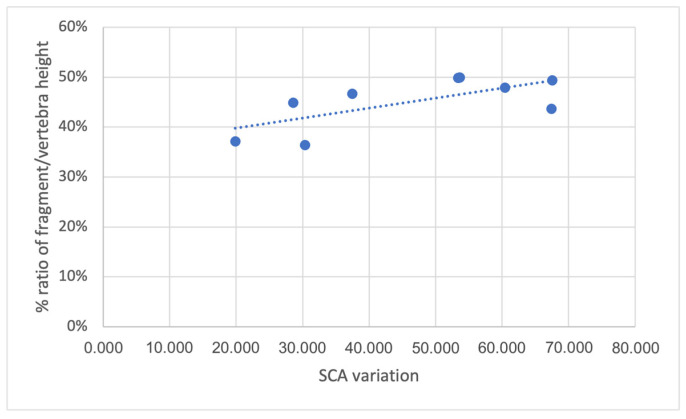
Dispersion graph of correlation between SCA variation and % ratio of fragment/vertebra height.

No significant association was found between delta SCA and delta LKA and the other radiological parameters (Table 2 and Figure 4), except for SCA improvement and % ratio of fragment/vertebra height. In this case, a greater SCA variation corresponded to a greater % ratio of fragment/vertebra height (rho = 0.682; *p* = 0.043).

No significant difference was observed in terms of SCA and LKA improvement among patients with the fracture level included in the fixation construct (Table 3).

All surgically treated patients showed improvement in terms of SCA and LKA values. Patients operated within 72 h showed a greater improvement of SCA and, in particular, of LKA values, compared to those treated later (>72 h) (Table 4).

## 4. Discussion

The optimal management of thoracolumbar burst fractures still remains controversial, despite the fact that multiple decision-making scores and algorithms have been proposed [5,6,7,10,11,13,14,15,16]. A subset of patients with A3 N0 M0 (according to the AO Spine Classification) [4] fail conservative treatment, leading to worsening pain, progressive kyphotic deformity, and potential neurological complications [7,16]. Traditionally, burst fractures with a TLICS score < 4 are suggested to be managed conservatively [10]. In our cohort (with a TLICS score of 2), surgical intervention is recommended, as per WFNS Spine guidelines for patients with TLICS < 4 who have intractable pain unresponsive to conservative measures or those requiring early mobilization due to high functional requirements [6]. Alan et al. also evaluated the role of LSC in guiding treatment decisions for neurologically intact patients with TLICS 4, finding LSC score to independently influence surgical strategy [11,13]. Patients in this “grey zone”, similar to our study, may exhibit a wide range of LSC score. The shift towards an earlier “upfront” interventional strategy is driven by institutional, surgeon-specific, and patient-specific factors; the aim is to alleviate axial mechanical pain, to prevent neurological deficits by indirectly restoring spinal canal area, and, above all, to allow a faster return to daily activities. Initial poor pain control, among other factors, is associated with the failure of conservative management; indeed, our patients presented with moderate-to-severe pain (NRS 6.22 ± 1.09) [7]. In candidates for surgery, a posterior approach, as used in the A3 burst fractures of our cohort, is considered effective to achieve these goals, even with LSC scores > 6 [6,11,17,18,19]. Including the fractured level in the fixation construct enhances its stability, avoiding long-segment fixations [6]. In our study, all patients were treated with short-segment construct, with the incorporation of the fractured level, when feasible, either mono- or bilaterally. However, the inclusion of the fractured vertebra did not significantly impact on SCA improvement (*p* = 0.905) or LKA restoration (*p* = 1.000).

Despite limited high-quality evidence supporting MIS techniques over the traditional open approach for thoracolumbar fractures, muscle sparing and reduced blood loss make them safe and effective, as performed in our cohort [6]. Additionally, fusion surgery was not performed due to the lack of compelling evidence suggesting kyphosis progression in non-fusion procedures, alongside the benefits of reduced blood loss and surgical time [6].

The posterior longitudinal ligament (PLL), extending from the foramen magnum to the L3-L4 intervertebral disc, plays a critical role in indirect decompression strategies such as ligamentotaxis. Of the two bundles that make it up, the shallow one has a width of 0.5–1 cm and the deep one, a segmental layer, with a width of 1 cm, has fibers extending laterally and outward to become wider and to fuse with the posterior annulus and postero-superior edge of the adjacent vertebral pedicle. Distally to L3, it remains only a slim structure [2,18]. The ligamentotaxis strategy is based on the potential reduction in posterior vertebral wall fracture fragment, increasing the tensile force in PLL fibers through lordosation and distraction maneuvers; the “repositio e vacuo” phenomenon achieved through the rapid volume/height increase in the fractured vertebral also contributes to indirect decompression [18]. Direct decompression is suggested in patients with neurological deficits, particularly in the presence of neurological impairment and radiological evidence of neural element compression. Additionally, cases involving epidural hematoma, which causes significant compression of the spinal cord or nerve roots, as well as those with severe radicular pain unresponsive to conservative management, may also require direct decompression to improve clinical outcomes [19,20]. For ligamentotaxis efficiency assessment, SCA is the parameter of choice, since the measurement of Mid-Sagittal Diameter (MSD) alone does not consider the morphological and positional variability of the fragment block in the spinal canal, also in light of the great gender and ethnicity differences in thoracolumbar vertebral size [2,18]. The effect of distraction, for the explained PLL anatomical configuration, is more evident for fragment blocks not located in lateral positions at the level of the spinal canal. In our cohort, the position of the fragment did not affect SCA improvement (*p* = 0.7261), even though in only one case the fragment was purely lateral.

The ligament’s integrity is crucial for effective decompression, with MRI being the gold standard for evaluating the posterior ligamentous complex (PLC) [18,21]. However, assessing PLC integrity remains challenging, with variable reliability among surgeons [22]. Schroeder and colleagues reported a poor reliability (k = 0.11) when more than 500 surgeons worldwide were asked to determine the integrity of the PLC in 10 cases of compression-type injuries [23]. Some studies have reported CT parameters especially of the fragment block for the indirect assessment of PLL integrity, with varying threshold values identified [2,24,25]. Generally, larger trapezoid-shaped fragments are difficult to reposition, especially with high-grade stenosis (>67%), suggesting PLL disruption or insufficiency [18,21]. In line with other studies, although in traumatic kinetics the rotation angles of the fragment block against the posterior vertebral wall (the vertebral body motion must always be considered three-dimensionally) are important determinants of fragment shape, in our cohort, the sagittal inversion (*p* = 0.0868) and horizontal rotation angle (*p* = 0.7116) had no significant impact on ligamentotaxis efficiency [2]. In contrast with other data in the literature, in our study, a higher % ratio of fragment/vertebra height was related to a better SCA improvement (*p* = 0.0432) [2]. A possible explanation may be that a wider sagittal area of the retropulsed posterior vertebral wall, with a sufficient and intact PLL, enables a wider distribution of distraction forces. The % ratio of fragment/vertebra width, instead, did not correlate with SCA restoration (*p* = 0.9959). This suggests that fragment width likely does not significantly affect reduction efficacy, possibly due to PLL anatomy and tensile force distribution, a relationship that we postulate for fragment height.

While robust evidence supporting early versus delayed treatment for thoracolumbar fractures with neurological impairment is scarce, also because major traumatic thoracolumbar fractures are commonly associated with polytrauma (with other implications), early surgery has been shown to be beneficial in cervical injuries for neurological recovery [26]. In neurologically intact patients with spinal fractures, early stabilization (<24 h) has been proposed to reduce major complications, aligning with “damage control” principles [27,28]. A metanalysis by Xing et al. recommends that patients with traumatic thoracolumbar fractures with/without SCI (spinal cord injury) may undergo surgery prior to 72 h to reduce hospitalization, intensive care unit length of stay, ventilator days, hospital expenses, and morbidity (and potentially mortality), particularly when the thoracic spine is involved; however, a definite conclusion cannot be drawn due to the heterogeneity of the included studies and the low level of evidence [29]. The underlying problem in obtaining high-quality evidence in this field is the difficult execution of randomized controlled trials to determine clinical outcomes and complications, because of the ethical concerns [29]. Moreover, given the fast recovery potentially achievable with this “early” strategy, also adopting mini invasive techniques, it is important to develop and apply Early Recovery After Surgery (ERAS) protocols to expedite safe discharge to home or outpatient physiotherapy [30].

On the contrary, conclusive data regarding optimal timing for an optimal effective surgical indirect decompression via ligamentotaxis in thoracolumbar burst fractures without neurological deficits are lacking. Some reports suggest intervention within 3 days to optimize correct fracture healing [21]. A reasonable explanation of the importance of early surgery resides in the fact that it would facilitate the improvement of vertebral morphometric restoration by advancing the onset of increasingly robust healing mechanisms. Moreover, studies have yet to combine early versus late treatment comparison with the analysis of fragment block morphology in this highly selected “grey” population. All our surgically treated patients improved in terms of LKA and SCA. Notably, patients treated before 72 h presented a greater improvement in SCA (*p* = 0.016) and, in particular, LKA (*p* = 0.004) values compared to those treated later. The 72 h cutoff used in our study is arbitrary and warrants further investigation. Although early stabilization has been described as ranging anywhere from 8 to 72 h, the often assumed 72 h stratification point is based on preclinical studies on murine models, which demonstrated that the early decompression of acute SCI results in improved neurological recovery [31].

The limitations of our study include its retrospective design and small, highly selective sample size, limiting generalizability. The lack of a conservative treatment/alternative therapy/control group, the short follow-up duration, and supine imaging evaluation without accounting for local and regional sagittal/coronal variations in upright standing are important additional constraints. Furthermore, physiological bone turnover processes affecting incompletely reduced fragment blocks may further influence SCA improvements. We think that this study could represent a basis for adequate prospective multicentric studies employing rigorous methods and a larger cohort of patients, all of which are necessary to confirm or contrast these findings, mitigating limitations.

## 5. Conclusions

In patients with single traumatic A3N0M0 thoracolumbar fractures, early (<72 h) short-segment percutaneous fixation can be considered, barring clear contraindications, particularly in young adult patients with high functional demands and moderate-to-severe axial pain. This early intervention strategy appears to correlate with enhanced indirect decompression and the restoration of the local kyphotic angle (LKA). Moreover, in patients with intact posterior longitudinal ligament (PLL), a larger fragment block relative to the posterior vertebral height (PVH) of the intact adjacent vertebrae appears to be associated with greater improvement in the spinal canal area (SCA).

## Figures and Tables

**Table 1:** Patient, injury mechanism, surgery, and radiological fragment block characteristics.

N	
**Patient sample**	
Sex (M), n (%)	5 (55.56%)
Age (years), mean ± SD	52.22 ± 13.73
BMI (Kg/m^2^), mean ± SD	27.66 ± 3.96
Fracture level, n (%)	
L1	8 (88.89%)
L2	1 (11.11%)
Mechanism of injury, n (%)	
Fall > 3 m	7 (77.78%)
Road traffic accident	2 (22.22%)
NRS	
Pre-operative, mean ± SD	6.22 ± 1.09
Post-operative, mean ± SD	2.22 ± 1.20
Delta, mean ± SD (95%CI)	−4 ± 0.87 (−4.67; −3.33)
Length of stay, mean ± SD	7.33 ± 5.45
**Surgical details**	
Fixed levels	
Fracture site not involved	4 (44.44%)
Fracture site—1 screw	1 (11.11%)
Fracture site—2 screws	4 (44.44%)
Surgical time (minutes), mean ± SD	109.22 ± 16.69
Timing (>72 h), n (%)	4 (44.44%)
**Fragment block parameters**	
IA	
Pre, mean ± SD	24.34 ± 11.16
Post, mean ± SD	18.97 ± 12.07
Delta, mean ± SD (95%CI)	−5.37 ± 4.97 (−9.19; −1.55)
*p* pre–post	0.004
HRA	
Pre, mean ± SD	10.19 ± 5.30
Post, mean ± SD	6.19 ± 4.75
Delta, mean ± SD (95%CI)	−4.00 ± 3.61 (−6.77; −1.22)
*p* pre–post	0.004
% ratio of fragment/vertebra height, mean ± SD	45.1 ± 5.3 (41.0; 49.2)
% ratio of fragment/vertebra width, mean ± SD	61.0 ± 18.8 (46.5; 75.5)

**Table 2:** Statistical correlation between fragment block (radiological parameters and position) and SCA (spinal canal area) variation or LKA (local kyphotic angle) variation.

	Delta SCA		Delta LKA	
	rho	*p*	rho	*p*
IA pre	−0.6012	0.0868	0.3969	0.2902
HRA pre	0.1440	0.7116	−0.1248	0.7491
**% ratio of fragment/vertebra height**	0.6816	**0.0432**	−0.5527	0.1227
% ratio of fragment/vertebra width	−0.0020	0.9959	−0.1233	0.7521
Fragment Position		0.7261		0.7261
Lateral	53.6		−3.96	
Paramedian	29.8 ± 15.5		−3.92 ± 1.68	
Median	38.6 ± 25.4		−2.61 ± 1.78	

**Table 3:** Statistical correlation between the inclusion/non inclusion of the fractured level in the fixation construct and SCA (spinal canal area) variation or LKA (local kyphotic angle) variation.

	Fracture Level Included	Fracture Level Not Included	*p*
N	5	4	
LKA delta	−3.61 ± 2.08	−3.34 ± 1.10	1.000
SCA delta	46.7 ± 13.2	46.3 ± 24.8	0.905

**Table 4:** Statistical correlation between the timing of surgery and the SCA (spinal canal area) or LKA (local kyphotic angle) variation.

	Timing < 72 h	Timing > 72 h	*p*
N	5	4	
SCA			
Pre, mean ± SD	234.2 ± 41.2	249.6 ± 32.5	0.562
Post, mean ± SD	294.6 ± 46.1	278.6 ± 27.9	0.564
**Delta, mean ± SD (95%CI)**	**60.5 ± 7.0 (51.8; 69.1)**	**29.0 ± 7.2 (17.5; 40.6)**	**0.016**
LKA			
Pre, mean ± SD	11.71 ± 5.62	13.73 ± 0.65	0.504
Post, mean ± SD	7.06 ± 5.52	11.68 ± 1.05	0.147
**Delta, mean ± SD (95%CI)**	**−4.65 ± 1.06 (−5.97; −3.33)**	**−2.04 ± 0.70 (−3.16; −0.93)**	**0.004**

## Data Availability

The data that support the findings of this study are not openly available due to reasons of sensitivity but they are available from the corresponding author upon reasonable request.

## Abbreviations

SCA: spinal canal area; LKA: local kyphotic angle; TL: thoracolumbar; SAVES: Spinal Adverse Events Severity System; TLICS: Thoracolumbar Injury Classification System; LSC: Load-Sharing Classification; CT: computed tomography; MRI: magnetic resonance imaging; PLC: posterior ligamentous complex; AVH: anterior vertebral height; PVH: posterior vertebral height; IA: inversion angle; HRA: horizontal rotation angle; PLL: posterior longitudinal ligament; LOS: length of stay; UTI: urinary tract infection; SIADH: syndrome of inappropriate antidiuretic hormone; NRS: Numeric Rating Scale.
